# Small RNA Barcodes for Sensitive Labeling of AAV Transcripts

**DOI:** 10.1523/ENEURO.0034-26.2026

**Published:** 2026-08-04

**Authors:** Yessenia Magaña, Shanshan Wang, Albertina Torreblanca-Zanca, Lai Wei, Michael J. Castle

**Affiliations:** ^1^Departments of Neurosciences, University of California – San Diego, La Jolla, California 92093; ^2^Anesthesiology, University of California – San Diego, La Jolla, California 92093; ^3^Pediatrics, University of California – San Diego, La Jolla, California 92093

**Keywords:** AAV, adeno-associated virus, BaseScope, in situ hybridization, RNA

## Abstract

Adeno-associated virus (AAV) vectors are widely used for neuroscience research. Labeling AAV mRNA by in situ hybridization can track AAV transduction with high sensitivity and without the need for reporter genes. However, it is challenging to detect similar mRNA transcripts when multiple AAVs are injected into the same animal, or when a single AAV produces multiple distinct transcripts. To address these challenges, we developed methods for sensitive labeling of small barcodes on AAV mRNA at single-transcript resolution*.* These small RNA barcodes (ranging from 40 to 44 bases) can be easily added to any AAV vector genome. We provide detailed step-by-step protocols for sensitive and flexible labeling of barcoded AAV mRNA by BaseScope in situ hybridization. We validated these labeling methods in male and female mice and in a female rhesus macaque. RNA barcoding can improve the reliability of multiplexed in vivo screening: by adding one barcode to half of the AAVs in a pooled library and a second barcode to the other half, barcode labeling can identify cells that receive multiple AAVs without the need for reporter proteins. This approach is also useful for intersectional experiments that combine multiple AAV vectors: multiple unique barcodes can track the spatial distribution of each AAV and can separately label transcripts from different AAV genome conformations, such as before or after Cre-Lox recombination. These new methods for sensitive multiplexed labeling of AAV mRNA will support greater rigor and reliability in neuroscience research.

## Significance Statement

Adeno-associated virus (AAV) vectors are commonly used in neuroscience research. Modern experimental designs are increasingly complex, often injecting two or more AAVs into different regions, or screening a library of many unique AAVs injected together into a single animal. In such complex experiments, it is challenging to track spatial distribution and rates of coinfection among multiple AAV vectors, especially if the AAVs do not carry reporter genes. To address these challenges, we developed small RNA barcodes and methods for highly sensitive labeling of barcoded AAV mRNA. These small barcodes can be easily added to any AAV genome without altering the experimental design, supporting the assessment of injection targeting and coinfection rates among multiple AAVs without the need for reporter genes.

## Introduction

Adeno-associated virus (AAV) vectors can drive strong, safe, and long-lasting gene expression in nervous system cells (Murlidharan et al., 2014; Castle et al., 2016; Deverman et al., 2018). AAV vectors are widely used to overexpress recombinant genes and to silence endogenous genes for both neuroscience research and neurological gene therapy (Haggerty et al., 2020; Ling et al., 2023).

An emerging technique in neuroscience research is multiplexed in vivo screening of many candidate AAV vectors packaged together as a single pooled AAV library (Bock et al., 2022; Ramani et al., 2025; Zheng et al., 2025). Genes that exacerbate or protect against a biological perturbation or disease model can be identified by screening a library of many AAVs that each overexpress or silence a different gene. To independently assess the impact of each genetic manipulation, it is important to maximize the number of cells that receive only a single AAV from the library. The dose at which most cells receive only a single AAV is typically determined by coinjecting two or three AAVs carrying reporter genes. The percentage of cells that express only one reporter are then quantified by immunolabeling or flow cytometry (Zheng et al., 2025). However, these techniques may underestimate the number of cells that receive multiple AAVs due to insufficient detection sensitivity, and mixed reporter AAVs may misrepresent the rate of coinfection by the experimental AAV library.

Intersectional experiments that combine multiple AAV vectors to target defined neuron populations are also increasingly popular in neuroscience research (Haggerty et al., 2020; Weinholtz and Castle, 2021). For intersectional expression, often a first “AAV-Cre” encodes Cre recombinase, while a second “Cre-dependent AAV” carries an inverted transgene flanked by Cre-recognition “lox” sites (Haggerty et al., 2020; Weinholtz and Castle, 2021). When both AAVs transduce the same cell, Cre recombines the lox sites, which flips the transgene into the correct orientation and activates gene expression only in cells that receive both vectors (Sauer and Henderson, 1988; Tsien et al., 1996). Delivering AAV-Cre by retrograde transport can specifically activate gene expression in neurons that project to a defined brain region, while delivering AAV-Cre by transsynaptic anterograde transport can activate expression only in neurons that receive inputs from a defined region (Tervo et al., 2016; Zingg et al., 2017). This is a powerful approach for targeted manipulation and tracing of neural circuits, but results may be biased by variable targeting of either AAV injection, by spontaneous recombination of the Cre-dependent vector, or by “leaky” transcription of the inverted transgene in the absence of Cre (Fischer et al., 2019). It is challenging to examine these potential confounds by standard immunolabeling techniques, resulting in uncertainty regarding the targeting and spread of each AAV, the percentage of the target neurons that receive each AAV, and whether there is leaky expression of the Cre-dependent AAV without Cre.

To address these challenges in modern neuroscience research, we developed small RNA barcodes and protocols for highly sensitive labeling of AAV transcripts using the BaseScope in situ hybridization platform. Built on the previously established RNAscope technology, BaseScope uses “Z” probes that each comprise ∼20 bases complementary to the target RNA sequence with a 14-base tail for signal amplification (Wang et al., 2012; Baker et al., 2017). Because signal amplification requires two *Z* probes to bind adjacent RNA sequences, off-target binding of a single *Z* probe does not produce labeling (Wang et al., 2012). This greatly improves specificity over traditional in situ hybridization methods, allowing for extensive signal amplification without off-target labeling. BaseScope can thus specifically detect a minimal 40-base RNA barcode using only one pair of *Z* probes (a “1ZZ” probe). These small barcodes can be readily added to any AAV vector genome. Barcode labeling can be performed on formaldehyde-fixed tissue samples and does not require special handling of tissue. We provide detailed step-by-step protocols for BaseScope labeling of fixed frozen nervous tissue as Extended Data.

Beyond more sensitive and flexible labeling of AAV transcripts, these small RNA barcodes and in situ hybridization methods can track the distribution of multiple AAVs, separately label transcripts from different AAV genome conformations (such as before or after Cre-Lox recombination), and identify cells that exhibit leaky expression from Cre-dependent AAVs in the absence of AAV-Cre. Barcodes can also be added to AAV libraries to detect cells transduced by multiple AAVs with maximum sensitivity and without the need for reporter proteins. This new approach for sensitive multiplexed labeling of AAV transcripts will support improved rigor and reproducibility in modern neuroscience research.

## Materials and Methods

### AAV vectors

AAV genome plasmids were cloned by Gibson assembly (Gibson et al., 2009). The AAV-CAG-smFLAG-WPRE and AAV-CAG-sfGFP-WPRE plasmids both comprised two 179 bp inverted terminal repeats (ITRs) from the AAV2 genome flanking (from 5′ to 3′): the 1.67 kb chicken beta-actin gene (CAG) promoter (Niwa et al., 1991), the protein coding sequence, the RNA barcode 1 sequence in the 3′ untranslated region (3′UTR) immediately following the protein coding sequence (CCCAGTCAGCAGCCCTGTTCACTGCCCTAAGCCCTCTAGC), the 589 bp woodchuck hepatitis virus posttranscriptional regulatory element (WPRE; Zufferey et al., 1999), the 224 bp bovine growth hormone polyadenylation (bGH polyA) signal (Pfarr et al., 1986), and the 177 bp toll-like receptor 9-inhibitory oligonucleotide (io2) sequence (Chan et al., 2021). The protein coding sequence encoded either smFLAG, an engineered variant of mRuby2 with 10 FLAG epitopes for immunolabeling (Viswanathan et al., 2015), or superfolder GFP (sfGFP; Pédelacq et al., 2006). The self-complementary AAV (scAAV) hSyn1-BDNF (brain-derived neurotrophic factor) plasmid comprised a 107 bp truncated 5′ AAV2 ITR lacking a terminal resolution sequence for double-stranded genome packaging (McCarty et al., 2001) and a full-length 3′ AAV2 ITR, flanking (from 5′ to 3′): the 469 bp human synapsin 1 (hSyn1) promoter (Kügler et al., 2003), a 110 bp synthetic intron, the 774 bp human pre-pro-BDNF protein coding sequence, the RNA barcode 1 sequence in the 3′UTR immediately following BDNF, and the 477 bp human growth hormone (hGH) polyA signal. The synthetic intron combined strong mammalian consensus splice donor, splice acceptor, and branch point sequences: CCTAAAACAGGTAAGTCCCATTTGTACAAATCAGGTCTGAAGAGTTTACTTAATTCCTCTACTAACCTTGTTCATCTTTTCTTTTTTTTTCTACAGGTCCT GGGCCGTAT.

The AAV-hSyn1-Cre-WPRE plasmid comprised two AAV2 ITRs flanking (from 5′ to 3′): the hSyn1 promoter, the 110 bp synthetic intron, the 1.05 kb codon-optimized Cre recombinase protein coding sequence with an N-terminal nuclear localization signal (Shimshek et al., 2002), the RNA barcode 3 sequence in the 3′ untranslated region (3′UTR) immediately following Cre (TGACTTTGGCAATCCCTTGTGTCTCTCTGTATAGGCTGGCTCAA), the WPRE, the bGH polyA signal, and the io2 sequence. The Cre-dependent AAV-Cbh-FLEX-tdTomato-WPRE plasmid comprised two AAV2 ITRs flanking (from 5′ to 3′): the io2 sequence, the 800 bp chicken beta-actin hybrid intron (Cbh) promoter (Gray et al., 2011), a flip-excision (FLEX) cassette comprising two inversely oriented pairs of LoxP and Lox2272 sites on either side of the Cre-dependent sequence (see below; Atasoy et al., 2008), the WPRE, and the hGH polyA signal. The Cre-dependent sequence comprised (from 5′ to 3′): the RNA barcode 1 sequence, an inverted RNA barcode 2 sequence (AATTACAAGCCTACCTGTGGCTAATAGGCCAAGCAGTTGTTGGT), and an inverted tdTomato coding sequence (Shaner et al., 2004). This Cre-dependent vector used an “ATG-out” design to ensure that translation of tdTomato occurred only after recombination by Cre: the tdTomato gene lacked an ATG start codon, and a single ATG start codon was instead placed between the Cbh promoter and the lox sites (Fischer et al., 2019).

To generate the AAV guide RNA (gRNA) plasmid library, two backbone plasmids were cloned by modifying the AAV-hSyn1-Cre-WPRE plasmid (above) to carry either RNA barcode 1 or RNA barcode 2 in the 3′UTR. Five individual gRNA expression constructs targeting PTEN mRNA and four constructs targeting SOCS3 mRNA were generated by Golden Gate assembly. These gRNA constructs were pooled at equal ratios, amplified by PCR, and simultaneously inserted into both AAV-Cre backbone plasmids using a single Gibson assembly reaction. Gibson products were transformed into chemically competent *E. coli* (New England Biolabs, C2987H) for initial verification by restriction digest and plasmid sequencing. The same Gibson reaction was then electroporated into electrocompetent cells (New England Biolabs, C3020), and the final plasmid library was produced using an EndoFree Plasmid Maxi Kit (Qiagen). The final library was validated by restriction digest and Oxford Nanopore sequencing (Plasmidsaurus) to confirm full-length vector recovery, gRNA diversity, and barcode distribution. Each plasmid in the library comprised two AAV2 ITRs flanking (from 5′ to 3′): the 250 bp human U6 promoter (Goomer and Kunkel, 1992), one of five gRNA targeting PTEN mRNA, a second U6 promoter, one of four gRNA targeting SOCS3 mRNA, the hSyn1 promoter, the synthetic intron, the Cre recombinase protein coding sequence, either RNA barcode 1 or RNA barcode 2, the WPRE, the bGH polyA signal, and the io2 sequence. This resulted in 40 total plasmids: 20 unique gRNA combinations with RNA barcode 1 in the 3′UTR and the same 20 gRNA combination with RNA barcode 2 in the 3′UTR.

The AAV-gRNA vector library was packaged into the AAV2retro capsid by PackGene Biotech. All other AAV vectors were produced by VectorBuilder. All AAVs were produced by triple transfection of HEK293 cells and cesium chloride gradient purification (Ayuso et al., 2010). Titers were determined by quantitative PCR (Sanmiguel et al., 2019). AAVs were buffered in 0.01 M phosphate-buffered saline (PBS), pH 7.4, supplemented with 200 mM NaCl and 0.001% pluronic F-68. Immediately prior to injection, AAVs were diluted to the desired concentration using sterile 0.01 M PBS supplemented with 0.005% pluronic F-68.

### Animal procedures

All mouse procedures were performed in accordance with the Department of Veterans Affairs San Diego Healthcare System animal care committee's regulations. Mice were deeply anesthetized by inhalation of 2% isoflurane in 100% oxygen at a flow rate of 1.5 L/min throughout all surgical procedures. AAV injections were performed using a pulled glass micropipette mounted on a stereotactic frame and connected with vacuum tubing to a Picospritzer II pressure injector (General Valve). Micropipettes had an outer diameter of 35–45 µm (Lu et al., 2019). AAV was injected by repeated pressure pulses at a pressure of 20 PSI and a pulse duration of 5–8 ms.

Two adult male C57/BL6J mice were treated by intracortical injection of AAV9-CAG-smFLAG at a concentration of 5 × 10^12^ vector genomes (vg)/ml. A craniotomy was performed, and AAV9 was injected into eight sites on each side of the brain at the following coordinates relative to bregma: A/P 1.8 mm, M/L ±1 and 1.5 mm; A/P 0.5 mm, M/L ±1.2, 1.5, and 2.2 mm; A/P 0.0 mm, M/L ±1.2, 1.5, and 2.2 mm. A volume of 0.1 µl was injected into each site at a depth of 0.5 mm beneath the cortical surface. Each injection was performed over 30 s, followed by a dwell time of 10 s before withdrawing the micropipette from the brain. A total dose of 8 × 10^9^ vg was administered to each mouse (4 × 10^9^ vg per side). Two adult female C57/BL6J mice were treated by intracortical injection of AAV9-CAG-sfGFP at a concentration of 5 × 10^12^ vg/ml, using methods identical to the above.

Four adult male C57/BL6J mice were treated by intracortical injection of AAV9-Cbh-FLEX-tdTomato at a concentration of 1 × 10^13^ vg/ml. The surgical procedures were identical to those described above, but a volume of 0.15 µl was injected at each site, resulting in a total dose of 2.4 × 10^10^ vg per mouse (1.2 × 10^10^ vg per side). During the same surgical session, two of these *N* = 4 mice were also treated by intraspinal injection of AAV2retro-hSyn1-Cre at a concentration of 1 × 10^13^ vg/ml. A laminectomy was performed to expose the C7 spinal cord. The dura was punctured using a 27-gauge needle, and AAV2retro was injected at three sites evenly spaced across the C7 spinal segment and 0.4 mm lateral of spinal midline. At each site, the micropipette was lowered to 0.5 mm beneath the spinal surface and 0.2 µl of AAV2retro was injected. The micropipette was then raised to 0.3 mm beneath the spinal surface and another 0.2 µl was injected. Each injection was performed over 1 min, followed by a dwell time of 30 s. The same three sites were then injected on the other side of the spinal cord, resulting in a total dose of 2.4 × 10^10^ vg per mouse (1.2 × 10^10^ vg per side).

Twelve adult male C57/BL6J mice were treated by intraspinal injection of the AAV2retro-gRNA library. Half of these mice (*N* = 6) were treated using procedures identical to those described above, with injections of 0.4 µl split between two depths at three sites on each side of C7 spinal cord, for a total volume of 1.2 µl per side. Three different concentrations of AAV2retro were injected (*N* = 2 mice each): 7.4 × 10^10^ vg/ml (8.9 × 10^7^ vg per side), 2.5 × 10^10^ vg/ml (3.0 × 10^7^ vg per side), and 8.2 × 10^9^ vg/ml (9.8 × 10^6^ vg per side). The remaining *N* = 6 mice were treated using similar procedures, but with two additional sites per side targeting C6 spinal cord: injections of 0.4 µl were split between two depths at five sites on each side of C6 and C7 spinal cord, for a total volume of 2.0 µl per side. Three different concentrations of AAV2retro were injected (*N* = 2 mice each): 5 × 10^10^ vg/ml (1 × 10^8^ vg per side), 3 × 10^10^ vg/ml (6.0 × 10^7^ vg per side), and 1.5 × 10^10^ vg/ml (3.0 × 10^7^ vg per side).

Following each surgery the skin was secured using surgical staples. Standard postoperative care included daily injections of lactated Ringer's solution, banamine, and ampicillin for 3 d after surgery. Four weeks after surgery, mice were transcardially perfused with nuclease-free 0.01 M PBS followed by 4% paraformaldehyde (PFA). Brains were drop fixed in 4% PFA for ∼24 h at 4°C, then cryoprotected by immersion in 30% sucrose at 4°C until sectioning.

All macaque procedures were performed in accordance with the University of California, Davis Animal Care Committee's regulations. A 12-year-old female rhesus macaque monkey was treated by AAV infusion into cisterna magna as previously described (Samaranch et al., 2012, 2019). Under anesthesia, the subject's head was placed in a stereotactic frame, a sterile 25-gauge needle mounted on a micromanipulator was advanced 4 mm into cisterna magna, and 3.3 ml of cerebrospinal fluid was withdrawn through sterile microbore tubing at a rate of 0.3 ml per minute using a syringe pump (Legato 130, KD Scientific). A syringe containing scAAV-hSyn1-BDNF was attached to the tubing, and 2.8 ml containing 6 × 10^13^ vg was infused at a rate of 0.2 ml per minute. The line was then flushed with 0.5 ml of 0.01 M PBS at a rate of 0.2 ml per minute. After 10 min dwell time, the needle was slowly withdrawn from cisterna magna and standard postoperative care was administered. After 3 months, the subject was transcardially perfused with nuclease-free 0.01 M PBS. The spinal column was dissected, the dorsal bone was removed to expose the spinal cord, and the spinal column was immersed in 4% PFA for ∼48 h at 4°C. The spinal cord was then dissected and cryoprotected by immersion in 10% glycerol, 2% dimethyl sulfoxide (DMSO) for ∼18 h at 4°C, followed by immersion in 20% glycerol, 2% DMSO at 4°C until sectioning.

### Histology

Tissues were frozen and sectioned using a sliding microtome set to a thickness of 40 µm. For immunofluorescent labeling, free-floating tissue sections were washed in Tris-buffered saline (TBS), incubated for 1 h at room temperature (RT) in blocking solution (5% donkey serum and 0.25% Triton X-100 in TBS), and incubated overnight at 4°C in blocking solution containing primary antibodies. Sections were then washed in TBS, incubated for 2 h at RT in blocking solution containing 4 µg/ml of secondary antibodies conjugated to Alexa Fluor (AF) fluorescent dyes AF488, AF568, or AF647 (Jackson ImmunoResearch), washed in TBS, mounted on gelatin-coated positively charged slides, and coverslipped in Mowiol 4-88 mounting medium (MilliporeSigma 9002-89-5). Primary antibodies used in this study included the following: 0.14 µg/ml of monoclonal rabbit anti-FLAG tag (Cell Signaling 14793), 1 µg/ml of polyclonal rabbit anti-mCherry (EnCor Biotechnology RPCA-mCherry), a 1:2,000 dilution of polyclonal guinea pig anti-NeuN (MilliporeSigma abn90), and 0.6 µg/ml of polyclonal rabbit anti-BDNF (Chicago Proteintech, custom antibody preparation against human pre-pro-BDNF carrying an N-terminal glutathione S-transferase tag). For immunolabeling of BDNF, prior to the protocol above, spinal cord sections were washed in TBS, incubated in 0.01 M Tris-HCl (pH 9.0) at 80°C for 20 min, and incubated in fresh 2% PFA and 0.2% para-benzoquinone solution for 5 min at RT.

BaseScope in situ hybridization was performed following the step-by-step protocols provided as Extended Data Figures 1-1 and 3-1. The following probes were used: BaseScope barcode 1 probe, channel 1 (Advanced Cell Diagnostics 902371-C1), BaseScope barcode 2 probes, channel 1 and channel 2 (Advanced Cell Diagnostics 902341-C1 and 902341-C2), and BaseScope barcode 3 probe, channel 1 (Advanced Cell Diagnostics 1174221-C1).

### Image acquisition and analysis

All imaging was performed using a Zeiss Axio Scan Z1 slide-scanning microscope with 10× Plan-Apo objective (Zeiss 420640-9900-000) and Colibri 7 LED light source (Zeiss 423052-9770-000). All graphs were generated using GraphPad Prism software version 10.6.1.

To compare the number of distally transduced cells in thalamus detected by GFP immunolabeling or by barcode 1 RNA labeling, sagittal brain sections from two mice treated by cortical injection of AAV9-sfGFP were analyzed. Because each side of the brain received a separate injection of AAV9, each side was analyzed as an independent datapoint (*N* = 4 per condition). Matched sagittal sections (∼1.3 mm lateral of bregma) were stained by GFP immunofluorescence or by RNA barcode 1 in situ hybridization. For both conditions, one section was stained and one image was acquired from each side of each brain. Labeled cells in thalamus were manually counted using the Graphics module in ZEN Blue 3.1 software (Carl Zeiss). An unpaired two-tailed Mann–Whitney *U* test was performed to compare the number of labeled cells counted in each condition (*p* = 0.0286). The nonparametric Mann–Whitney *U* test was used because the sample size was not sufficient to determine whether the data were normally distributed.

To compare the number of transduced cells in the macaque spinal cord detected by BDNF immunolabeling or by barcode 1 RNA labeling, transverse L5 lumbar spinal cord sections from a rhesus macaque treated by cisterna magna infusion of scAAV9-BDNF were analyzed. Anatomically matched sections were stained by BDNF immunofluorescence or by RNA barcode 1 in situ hybridization, and images of each section were acquired (*N* = 5 per condition). For each image, labeled cells in both the left and right ventral horns were manually counted using the Graphics module in ZEN Blue 3.1 software (Carl Zeiss).

To quantify the number of single- and double-labeled cells in primary sensorimotor cortex after intraspinal injection of the AAV2retro-gRNA library, coronal sections (0.4–0.6 mm rostral of bregma) labeled by duplex BaseScope in situ hybridization for barcode 1 and barcode 2 were analyzed. Because in rodents corticospinal neurons almost exclusively project to the contralateral spinal cord (Friedli et al., 2015), and each side of the brain was thus separately targeted by AAV2retro injections on the corresponding side of the spinal cord (Nakashima et al., 2025), each side of the brain was analyzed as an independent datapoint (*N* = 4 per group). One image covering the entire unilateral primary sensorimotor cortex was exported and blinded from each side of each mouse. Blinding was performed by M.J.C. and quantification was performed by Y.M. while fully blinded to group identity. For each image, the numbers of single- and double-labeled neurons in sensorimotor cortex were counted using the Cell Counter plugin in FIJI (ImageJ) software version 2.17.0 (Schindelin et al., 2012).

## Results

### Validation of a small RNA barcode for sensitive labeling of AAV mRNA

RNA barcodes were sized between 40 and 44 bases to be compatible with a single 1ZZ BaseScope probe pair (Baker et al., 2017). The barcodes did not contain biologically active sequences such as start codons, CpG motifs, or consensus sequences for intron splicing or transcription initiation. RNA barcode probes were not predicted to cross-react with any known mammalian genome or RNA sequences by alignment search against taxID 40674 (mammals), with any known viral genome or RNA sequences (taxID 10239, viruses), or with any common synthetic sequences (taxID 32630, synthetic constructs; Camacho et al., 2009). We first validated a 40-base RNA barcode (“barcode 1”) across various AAV vectors, routes of delivery, and species (Figs. 1, 2). An AAV9 vector carrying RNA barcode 1 and the smFLAG reporter gene was injected into mouse sensorimotor cortex. Immunofluorescence for smFLAG labeled cells at the cortical injection site and axons throughout the brain that likely originated from cortical projection neurons (Fig. 1*A*). BaseScope in situ hybridization for barcode 1 strongly labeled AAV mRNA at the cortical injection site, with distribution similar to smFLAG (Fig. 1*A*). Beyond the injection site, cellular labeling of AAV mRNA was observed in the thalamus and midbrain, potentially indicating that distal neurons were transduced by axonal transport of AAV9 (Castle et al., 2014a,b). Few cells were labeled by smFLAG in these distal brain regions, suggesting that BaseScope labeling of barcoded AAV mRNA might more sensitively detect cells that are weakly transduced, such as those distal from the injection site. To examine this possibility, we directly compared the sensitivity of immunolabeling and in situ hybridization for quantifying distal transduction in thalamus after injection of barcoded AAV9-sfGFP into mouse sensorimotor cortex (Fig. 1*B*). Immunofluorescence for sfGFP extensively labeled corticothalamic axons projecting from the cortical injection site but only labeled 2.5 cell bodies per section in thalamus (Fig. 1*C*). In matched brain sections, in situ hybridization for RNA barcode 1 labeled 475.5 cells per section in thalamus (Fig. 1*C*). This suggests that thalamocortical neurons were extensively transduced by retrograde transport of AAV9 from the cortical injection site, but standard immunofluorescent labeling was not sufficiently sensitive to detect these weakly transduced neurons.

**Figure 1.**
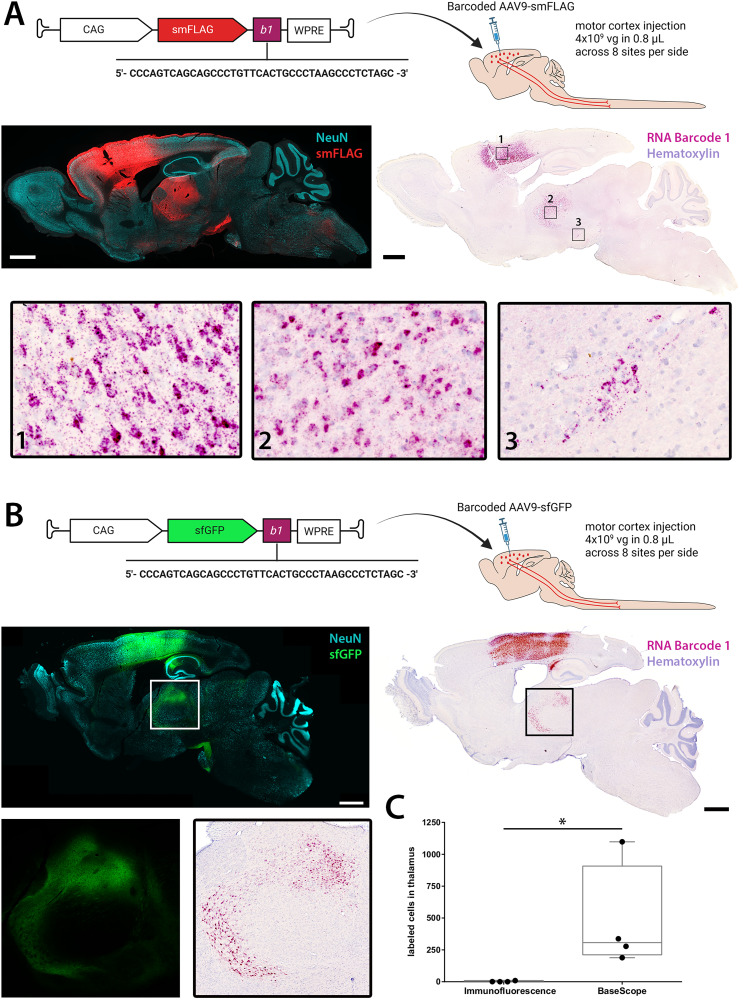
A small RNA barcode for sensitive labeling of AAV transcripts. ***A***, Four weeks after injection of barcoded AAV9-smFLAG into mouse sensorimotor cortex, BaseScope in situ hybridization for barcode 1 labeled AAV mRNA at the injection site (1) and also distal from the injection site in thalamus (2) and midbrain (3). BaseScope labeling was performed following the protocol provided as Extended Data Figure 1-1. Scale bars: 0.5 mm. ***B***, Four weeks after injection of barcoded AAV9-sfGFP into mouse sensorimotor cortex, sagittal brain sections were labeled by immunofluorescence for sfGFP (left) or by BaseScope in situ hybridization for AAV mRNA barcode 1 (right). Both methods labeled cells at the cortical injection site, but only in situ hybridization for mRNA barcode 1 extensively labeled cells in thalamus. Scale bars: 1 mm. ***C***, The number of cells in thalamus labeled by GFP immunofluorescence or by BaseScope in situ hybridization were quantified in 40-µm-thick brain sections. Each datapoint represents one side of the brain from one animal (*N* = 4 sides per condition). A mean of 475.5 cells per section were labeled by in situ hybridization, while a mean of 2.5 cells per section were labeled by immunofluorescence. Whiskers represent the minimum and maximum values. *p = 0.0286 (unpaired two-tailed Mann–Whitney *U* test).

10.1523/ENEURO.0034-26.2026.f1-1 Figure 1-1 BaseScope red protocol for fixed frozen CNS tissue. Download Figure 1-1, DOCX file.

**Figure 2.**
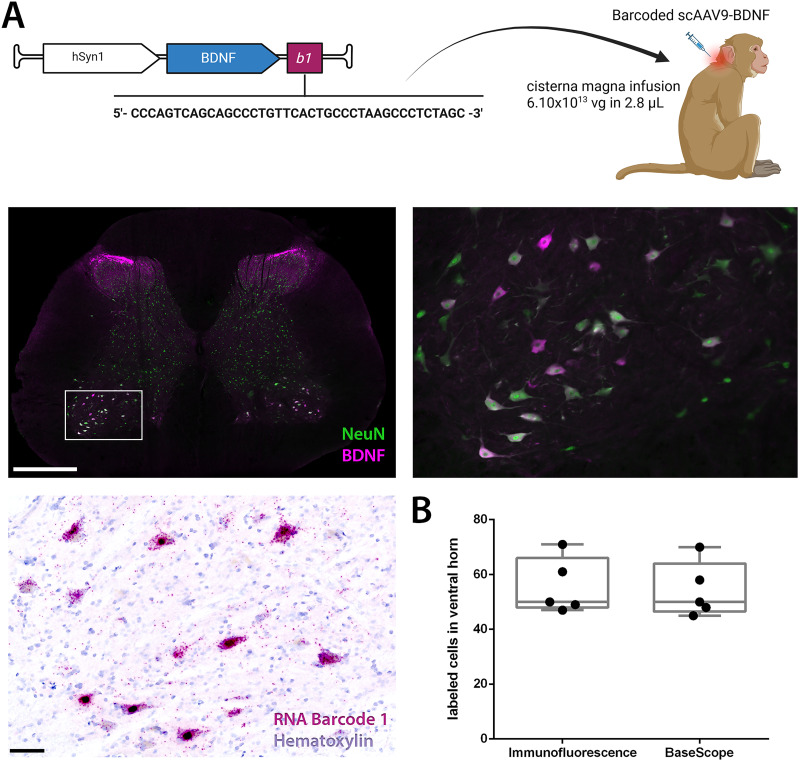
Labeling of barcoded AAV mRNA in the rhesus macaque spinal cord. ***A***, Three months after infusion of barcoded scAAV9-BDNF into the cerebrospinal fluid of an adult rhesus macaque monkey, BaseScope in situ hybridization labeled AAV mRNA in the ventral horn of L5 lumbar spinal cord. Scale bars: 0.1 mm (in situ hybridization), 0.5 mm (immunofluorescence). ***B***, The number of cells in the ventral horn of L5 spinal cord labeled by BDNF immunofluorescence or by BaseScope in situ hybridization were quantified in 40-µm-thick spinal cord sections. Each datapoint represents one spinal cord section (*N* = 5 sections per condition). A mean of 54.6 cells per section were labeled by in situ hybridization, while a mean of 55.6 cells per section were labeled by immunofluorescence. Whiskers represent the minimum and maximum values.

To validate barcode labeling across species, a self-complementary AAV9 vector carrying the BDNF gene and barcode 1 in the 3′UTR was infused into the cerebrospinal fluid of an adult female rhesus macaque (Fig. 2). Three months after treatment, immunolabeling of lumbar spinal cord detected BDNF expression in large ventral horn neurons (Fig. 2*A*), consistent with previous reports of gene delivery to spinal motor neurons after infusion of AAV9 into the primate cerebrospinal fluid (Gray et al., 2013; Meyer et al., 2015). However, BDNF immunolabeling may also detect endogenous BDNF protein, and cells transduced by AAV9 may secrete BDNF onto neighboring cells. BDNF immunolabeling thus may not accurately reflect the number of cells transduced by scAAV9-BDNF. To definitively determine the number of transduced cells, we performed BaseScope in situ hybridization for barcode 1. We observed labeling of AAV mRNA in a similar population of ventral horn neurons: immunofluorescence labeled 55.6 cells per section, while in situ hybridization labeled 54.6 cells per section (Fig. 2*B*). Barcode 1 labeling of AAV mRNA can thus ensure accurate quantification of AAV transduction when the AAV transgene product is secreted or is identical to an endogenous protein.

These results (Figs. 1, 2) demonstrate consistency of barcode 1 labeling across AAV vectors, routes of delivery, and species. In both the mouse brain and the macaque spinal cord, minimal nonspecific BaseScope labeling was observed outside the regions noted above, indicating that the barcode 1 probe is highly specific for AAV mRNA (Figs. 1, 2).

### Multiplexing RNA barcodes to detect cells transduced by single or multiple AAVs from a pooled AAV library

We validated a second RNA barcode (“barcode 2”), added both barcodes 1 and 2 to a pooled AAV library, and used barcode labeling to identify a surgical method and AAV dose that maximized the number of corticospinal neurons receiving only a single AAV from the library (Fig. 3). We produced an AAV2retro-gRNA library packaging 40 unique genomes: 20 gRNA with barcode 1 in the 3′UTR and 20 gRNA with barcode 2 in the 3′UTR. We then compared two surgical methods for injection of this pooled AAV2retro library into the mouse cervical spinal gray matter: AAV was injected into three sites per side across cervical level C7 or into five sites per side across cervical levels C6 and C7. For each surgical method, three decreasing doses of AAV2retro were tested. Duplex BaseScope in situ hybridization was then performed on coronal brain sections to determine the number of corticospinal neurons that were single- or double-labeled at each dose (Fig. 3*A*). Injection of the highest dose into three sites per side across C7 resulted in the greatest transduction of corticospinal neurons, with an average of 137.0 neurons transduced per side in a single 40-µm-thick section, but only 34.8% of transduced neurons were single labeled (Fig. 3*B*). Injection of the medium and low doses into three sites across C7 transduced fewer than 15 neurons per side. Injection of the highest dose into five sites across C6 and C7 resulted in transduction of 77.8 neurons per side, but again only 41.8% were single labeled. And again, injection of the low dose resulted in transduction of only six neurons per side. However, injection of the medium dose into five sites across C6 and C7 resulted in transduction of 43.5 neurons per side, 62.8% of which were single labeled. We thus identified a surgical method and AAV dose that maintains transduction strength while maximizing the number of corticospinal neurons that receive only a single candidate AAV from the pooled library.

**Figure 3.**
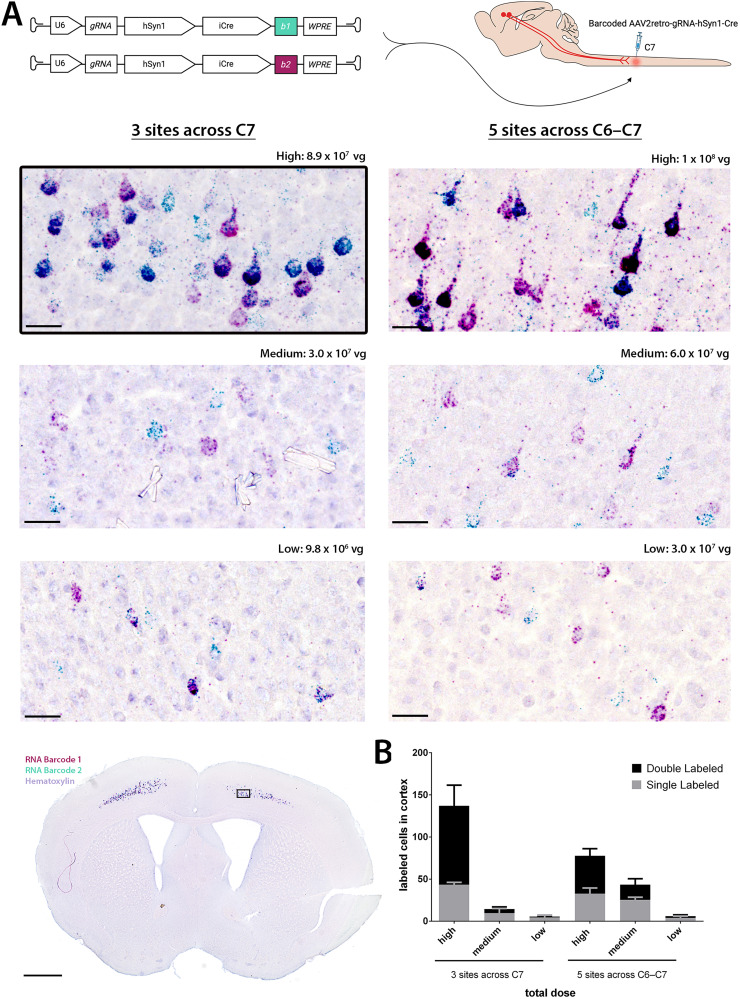
Multiplexed RNA barcoding of an AAV library for sensitive detection of cells transduced by single or multiple AAVs. An AAV2retro-gRNA library packaging 40 unique genomes (20 with RNA barcode 1 in the 3′UTR, and 20 with RNA barcode 2 in the 3′UTR) was injected into the mouse cervical spinal gray matter. Two surgical methods were compared: AAV2retro was injected into either three sites per side across cervical level C7 or into five sites per side across cervical levels C6 and C7. For each surgical method, three different doses of AAV2retro were tested. ***A***, Four weeks after treatment, duplex BaseScope in situ hybridization was performed on coronal brain sections to quantify the number of corticospinal neurons that were single- or double-labeled at each dose. Duplex BaseScope labeling was performed following the protocol provided as Extended Data Fig. 3-1. Scale bars: 0.5 mm (low magnification), 50 µm (high magnification). ***B***, After injection of AAV2retro into three sites per side across C7, at the highest dose an average of 137.0 neurons were transduced per side in a single 40-µm-thick section, but only 34.8% of transduced neurons were single labeled. At the medium and low doses, fewer than 15 neurons per side were transduced. After injection of AAV2retro into five sites per side across C6 and C7, at the highest dose an average of 77.8 neurons per side were transduced, but again only 41.8% were single labeled. However, at the medium dose 43.5 neurons per side were transduced and 62.8% were single labeled, providing reasonable strength of transduction and avoiding excessive overlap between multiple AAVs. *N* = 4 datapoints per dose. Error bars represent standard error of the mean.

10.1523/ENEURO.0034-26.2026.f3-1 Figure 3-1 BaseScope duplex protocol for fixed frozen CNS tissue. Download Figure 3-1, DOCX file.

### Multiplexing RNA barcodes to track Cre-dependent recombination and intersectional expression of AAV vectors

We validated an additional RNA barcode (“barcode 3”), and we multiplexed these three RNA barcodes to track the intersectional expression of two AAV vectors in the mouse brain. This experiment utilized a Cre-dependent AAV9-FLEX-tdTomato vector carrying two inversely oriented pairs of LoxP and Lox2272 sites surrounding: RNA barcode 1, an inverted RNA barcode 2 sequence, and an inverted tdTomato coding sequence (Atasoy et al., 2008; Fig. 4). Prior to recombination by Cre, RNA barcode 1 is present on the mRNA but the tdTomato sequence is inverted (no tdTomato is produced). After recombination by Cre, RNA barcode 2 and the tdTomato sequence are both present on the mRNA, and tdTomato is produced. This Cre-dependent AAV9-FLEX-tdTomato vector was injected into mouse sensorimotor cortex, and a second AAV2retro-Cre vector carrying barcode 3 was injected into the cervical spinal gray matter. Four weeks after treatment, tdTomato was specifically expressed in layer 5 corticospinal projection neurons (Fig. 4). We then performed duplex BaseScope in situ hybridization for barcode 1 and barcode 2 to track the distribution and Cre-dependent recombination of the AAV9-FLEX-tdTomato vector. Transcription of inactive AAV mRNA was observed in cells throughout sensorimotor cortex (barcode 1, green), reflecting the spread of unrecombined AAV9-FLEX-tdTomato surrounding the cortical injection site (Fig. 4*A*), while specific intersectional activation of AAV mRNA was observed in layer 5 corticospinal neurons (barcode 2, red; Fig. 4*A*). In negative control animals that did not receive AAV2retro-Cre, no tdTomato expression was detectable in cortex. Duplex BaseScope in situ hybridization again labeled inactive mRNA transcribed from unrecombined AAV9-FLEX-tdTomato genomes surrounding the cortical injection site (barcode 1, green). Without AAV2retro-Cre, no intersectional activation of AAV9-FLEX-tdTomato mRNA was detected in layer 5 corticospinal projection neurons (barcode 2, red). However, labeling of barcode 2 revealed sparse puncta throughout all cortical layers, potentially reflecting transcription of the antisense strand from unrecombined AAV genomes in cells that did not receive Cre (Fischer et al., 2019; Fig. 4*A*).

**Figure 4.**
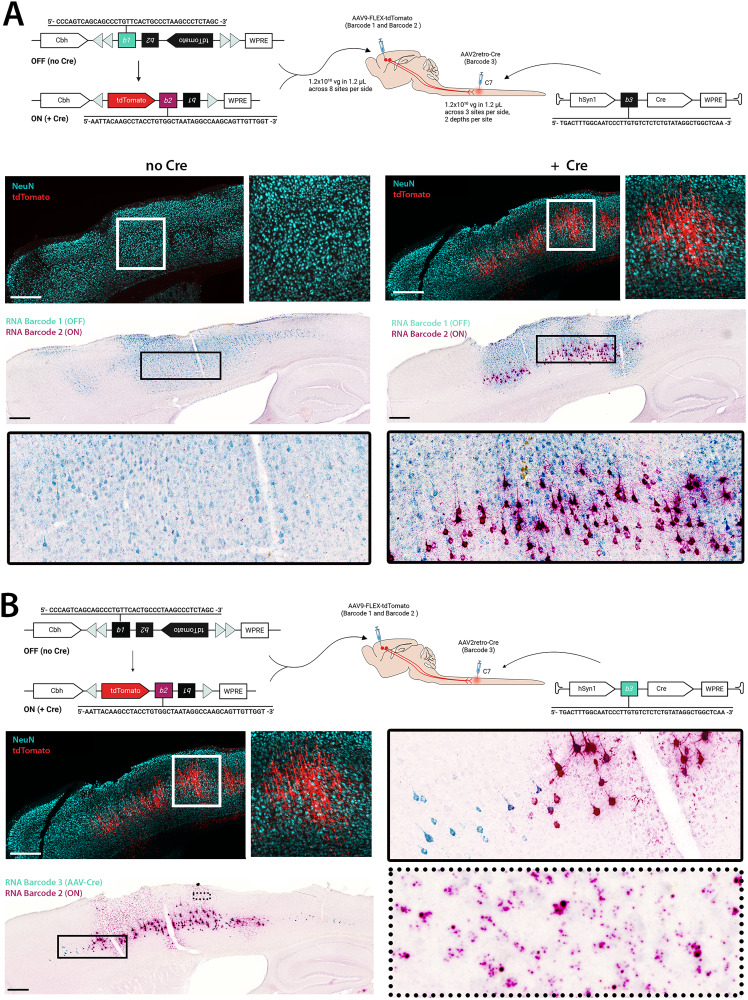
Multiplexed RNA barcodes track the Cre-dependent recombination of AAV genomes and the intersection of multiple AAV vectors. Cre-dependent AAV9-FLEX-tdTomato carrying barcode 1 (only active before Cre recombination) and barcode 2 (only active after Cre recombination) was injected into mouse sensorimotor cortex, and AAV2retro-Cre carrying barcode 3 was injected into the cervical spinal gray matter. ***A***, In negative control animals that did not receive AAV2retro-Cre, no tdTomato labeling was observed in cortex by immunofluorescence. Without Cre, duplex BaseScope in situ hybridization revealed transcription of inactive Cre-dependent AAV mRNA surrounding the cortical injection site (barcode 1, green). No intersectional activation of AAV mRNA was detected in layer 5 corticospinal projection neurons (barcode 2, red). In animals that received intraspinal injection of AAV2retro-Cre, intersectional expression of tdTomato in layer 5 corticospinal neurons was observed by immunofluorescence. Duplex BaseScope in situ hybridization again revealed transcription of inactive Cre-dependent AAV mRNA surrounding the cortical injection site (barcode 1, green), but after intraspinal injection of AAV2retro-Cre, extensive and specific intersectional activation of AAV mRNA was observed in layer 5 corticospinal neurons (barcode 2, red). Scale bars: 0.5 mm. ***B***, In animals that received intraspinal injection of AAV2retro-Cre carrying barcode 3, Duplex BaseScope in situ hybridization revealed both intersectional activation of AAV mRNA in layer 5 corticospinal neurons at the injection site (barcode 2, red) and extensive off-target labeling of mRNA carrying barcode 2 in other cortical layers. Specific transduction of layer 5 corticospinal neurons by AAV2retro-Cre (barcode 3, green) extended rostral and caudal of the cortical injection site, with no detectable barcode 3 labeling outside of layer 5, indicating that leaky expression of AAV mRNA carrying barcode 2 in other cortical layers was not caused by off-target expression of AAV2retro-Cre. Dense nuclear labeling of barcode 2 (red) likely reflects off-target binding of the barcode 2 probe to AAV DNA episomes, which can be prevented by DNase treatment prior to BaseScope staining (Extended Data Figs. 4-1, 4-2). Scale bars: 0.5 mm.

10.1523/ENEURO.0034-26.2026.f4-1 Figure 4-1 **The duration of heat-induced target retrieval impacts off-target BaseScope labeling of AAV DNA episomes.** Animals were treated by intraspinal injection of AAV2retro-Cre and intracortical injection of Cre-dependent AAV9-FLEX-tdTomato carrying barcode 1 (active before Cre recombination) and barcode 2 (active after Cre recombination) (Fig. 2). In these tissue sections, performing target retrieval in a steamer for 15 minutes at 99°C resulted in dense nuclear BaseScope labeling of both barcode 1 and barcode 2 across all cortical layers surrounding the injection site, consistent with labeling of AAV9-FLEX-tdTomato DNA episomes. Decreasing the target retrieval duration to 10 minutes greatly reduced labeling of episomal DNA, and also preserved strong labeling of active AAV mRNA in layer 5 corticospinal neurons (barcode 2, red). Further decreasing the target retrieval duration to 5 minutes further reduced labeling of episomal DNA, but also substantially reduced on-target labeling of active AAV mRNA in layer 5 (barcode 2, red). Scale bars: 0.2 mm (low magnification), 50 µm (high magnification). Download Figure 4-1, TIF file.

10.1523/ENEURO.0034-26.2026.f4-2 Figure 4-2 **DNase treatment prevents off-target BaseScope labeling of AAV DNA episomes.** Cre-dependent AAV9-FLEX-tdTomato carrying barcode 1 on the top strand (green) and barcode 2 on the bottom strand (red) was injected into mouse sensorimotor cortex. Mice did not receive AAV-Cre. RNase treatment greatly reduced the cytoplasmic labeling of AAV mRNA (barcode 1, green), but did not visibly decrease the dense nuclear labeling of both barcodes. DNase treatment did not visibly decrease the cytoplasmic labeling of AAV mRNA, but almost completely eliminated the dense nuclear labeling of AAV DNA episomes. No nuclear labeling of barcode 2 (red) was detected after DNase treatment. Scale bars: 0.5 mm (low magnification), 50 µm (high magnification). Download Figure 4-2, TIF file.

We also performed duplex BaseScope in situ hybridization for barcode 3 and barcode 2 to track the intersectional targeting of the AAV2retro-Cre and AAV9-FLEX-tdTomato vectors, respectively. Again, barcode 2 (red) strongly labeled active AAV9-Flex-tdTomato mRNA in layer 5 corticospinal neurons that received AAV2retro-Cre (Fig. 4*B*). Barcode 2 labeling was also observed across other cortical layers (Fig. 4*B*): this could reflect either off-target recombination of AAV9-FLEX-tdTomato by mistargeting of AAV2retro-Cre or leaky transcription of mRNA carrying barcode 2 from the antisense strand of unrecombined AAV9-FLEX-tdTomato genomes. Labeling of AAV2retro-Cre mRNA (barcode 3, green) was highly specific for layer 5 corticospinal neurons throughout sensorimotor cortex, with no detectable labeling of barcode 3 in other cortical layers (Fig. 4*B*). This indicates that AAV2retro-Cre was accurately targeted to layer 5 corticospinal projection neurons and that labeling of barcode 2 in other cortical layers likely reflects leaky transcription of mRNA from the antisense strand of unrecombined AAV9-FLEX-tdTomato genomes (Fischer et al., 2019).

### Additional validation of RNA barcode labeling

In RNAscope and BaseScope in situ hybridization protocols, heat-induced target retrieval is an essential step that reverses formaldehyde-induced crosslinking and unmasks mRNA for probe hybridization (Wang et al., 2012; Baker et al., 2017). When performing duplex BaseScope labeling after injection of AAV9 into mouse sensorimotor cortex (Fig. 4), following the manufacturer's recommendations for target retrieval (15 min at 99°C in a steamer) resulted in dense labeling of both barcode 1 and barcode 2 across all cortical layers surrounding the injection site (Extended Data Fig. 4-1). Rather than labeling mRNA as discrete puncta, this signal was densely packed into cell nuclei, consistent with labeling of the AAV9-FLEX-tdTomato DNA genome. After transduction, the AAV genome undergoes second-strand synthesis and persists in the nucleus as episomal double-stranded DNA (Afione et al., 1996). Because both barcode 1 and barcode 2 are present on double-stranded AAV9-FLEX-tdTomato DNA episomes, unmasking of AAV episomal DNA by extended target retrieval could potentially result in widespread labeling of cell nuclei by both probes. We hypothesized that a shorter duration of target retrieval would prevent unmasking of AAV DNA episomes and reduce off-target nuclear labeling. Decreasing target retrieval to 10 min at 99°C greatly reduced the dense nuclear labeling of AAV DNA, while preserving strong labeling of inactive AAV mRNA throughout cortex (barcode 1, green) and active AAV mRNA in layer 5 corticospinal neurons (barcode 2, red; Extended Data Fig. 4-1). Further decreasing target retrieval to 5 min at 99°C largely eliminated the nuclear labeling of AAV DNA but also substantially reduced on-target labeling of AAV mRNA (Extended Data Fig. 4-1).

To further investigate the potential labeling of episomal AAV DNA, we selectively removed DNA or RNA from tissue sections by DNase or RNase treatment prior to BaseScope in situ hybridization. We performed this experiment in mice that received the same Cre-dependent AAV9-FLEX-tdTomato vector used in Figure 4 but that did not receive an AAV-Cre vector. In the absence of Cre, few AAV genomes are expected to recombine: nearly all AAV mRNA should be transcribed from the top strand (barcode 1, green), and minimal AAV mRNA should be transcribed from the bottom strand (barcode 2, red). Strong cytoplasmic labeling of AAV mRNA transcribed from the top strand (barcode 1, green) was observed surrounding the cortical injection site, along with dense nuclear labeling of both barcodes consistent with off-target labeling of AAV DNA episomes (Extended Data Fig. 4-2). After RNase treatment, cytoplasmic labeling of AAV mRNA was greatly reduced (barcode 1, green), while the dense nuclear labeling was not visibly decreased (Extended Data Fig. 4-2). In contrast, DNase treatment almost completely eliminated the dense nuclear labeling (Extended Data Fig. 4-2). Minimal labeling of barcode 2 (red) was observed after DNase treatment. This provides further evidence that nuclear labeling was caused by off-target probe binding to AAV DNA episomes and demonstrates that off-target DNA labeling may be prevented by DNase treatment prior to BaseScope in situ hybridization.

We also examined the specificity of each barcode probe for the target RNA barcode sequence by labeling matched brain sections from mice that received AAV injection into motor cortex (Fig. 5). Matched sections were stained with each BaseScope barcode probe under identical conditions. Each probe specifically reacted with only the target RNA barcode and did not cross-react with other barcodes (Fig. 5).

**Figure 5.**
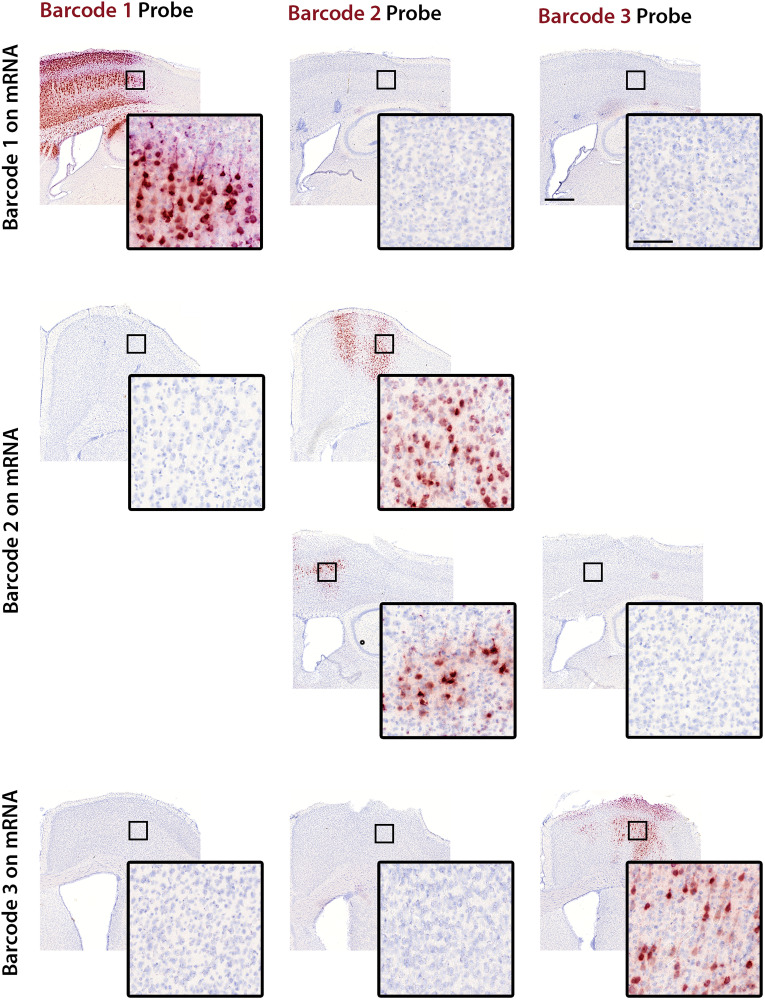
Barcode probes are specific for the target RNA barcode and do not cross-react. All images in each row are matched sections from the same mouse, stained under identical conditions with different BaseScope probes. All mice were treated by AAV injection into motor cortex (Fig. 1). In the first row, AAV mRNA carrying barcode 1 was not labeled by the barcode 2 or barcode 3 probes. In the second row, AAV mRNA carrying barcode 2 (and barcode 3) was not labeled by the barcode 1 probe. In the third row, AAV mRNA carrying barcode 2 (and barcode 1) was not labeled by the barcode 3 probe. In the fourth row, AAV mRNA carrying barcode 3 was not labeled by the barcode 1 or barcode 2 probes. Scale bars: 0.5 mm (low magnification), 100 µm (high magnification).

## Discussion

In this study, we present three small RNA barcodes and step-by-step protocols for sensitive and specific labeling of AAV mRNA across species. BaseScope in situ hybridization labeling of these new RNA barcodes has several advantages over standard immunolabeling techniques. First, this approach can detect mRNA from any AAV vector with single-transcript sensitivity, while the sensitivity of immunolabeling is highly dependent on the primary antibody and requires sufficient translation of the target protein (Wang et al., 2012). Second, these barcodes are smaller than 45 bases and can be readily added to any AAV genome, eliminating the need for epitope tags (which may interfere with normal protein function) or coexpression of reporter proteins (which increase mRNA transcript length and may reduce the expression of upstream genes). Third, the same validated barcodes can be used across experiments, eliminating the need to purchase and validate new primary antibodies or RNA probes for each target and improving consistency and reproducibility. Based on these advantages, we present several new applications for these RNA barcodes in modern neuroscience research.

The greater sensitivity of BaseScope in situ hybridization supports more accurate tracking of AAV distribution within the nervous system. After direct injection into the brain, AAV can enter axon terminals, undergo retrograde axonal transport, and transduce distal neurons that innervate the injection site (Paterna et al., 2004; Castle et al., 2014a). AAV can also undergo anterograde axonal transport and transsynaptically transduce distal brain regions innervated by neurons at the injection site (Castle et al., 2014a; Zingg et al., 2017). However, only a small fraction of injected AAV particles undergo axonal transport, and transduction of distal brain regions is typically much weaker than transduction at the injection site (Zingg et al., 2017). Transduction of distal brain regions by axonal transport thus may not be detectable by standard immunolabeling techniques. Indeed, the first reports of transsynaptic AAV anterograde transport used in situ hybridization to detect AAV mRNA in distal brain regions (Cearley and Wolfe, 2007; Castle et al., 2014a). In our current study, RNA barcode labeling revealed extensive transduction of thalamus after AAV9 was injected into mouse sensorimotor cortex, likely by retrograde transport along thalamocortical projections (Fig. 1). We also observed relatively sparse labeling of AAV mRNA in cells throughout midbrain, likely reflecting transduction by transsynaptic anterograde transport along projections from primary sensorimotor cortex to regions including substantia nigra and the ventral tegmental area (Fig. 1*A*; Muñoz-Castañeda et al., 2021). This highlights the importance of sensitive labeling for functional studies, as off-target expression of optogenetic ion channels, chemogenetic receptors, or other experimental genes by AAV axonal transport to connected brain regions could potentially bias results. Further, it may not be possible to track AAV transduction by immunolabeling when the AAV transgene product is secreted or is identical to an endogenous protein. We demonstrate that following treatment with an AAV vector encoding BDNF, which is secreted by transduced cells and identical to endogenous BDNF protein, labeling AAV mRNA can precisely track the number and distribution of transduced cells (Fig. 2).

Beyond improved tracking of AAV transcripts, RNA barcodes can also improve the reliability of multiplexed in vivo screening of pooled AAV libraries (Bock et al., 2022; Ramani et al., 2025; Zheng et al., 2025). When screening AAV libraries, it is essential that most cells receive only a single AAV in order to independently assess the impact of each vector (Zheng et al., 2025). The optimal dose and route of administration are typically determined by mixing and coinjecting two or three AAVs carrying reporter genes and then quantifying the percentage of cells that express only one reporter (Zheng et al., 2025). However, the rate of coinfection among these mixed reporter AAVs may not accurately predict the rate of coinfection from an entirely different pooled AAV library. In addition, immunolabeling of reporter proteins may underestimate the number of cells that receive multiple AAVs due to insufficient sensitivity. To address these concerns, we produced a pooled AAV library in which half of the AAVs carried RNA barcode 1 and the other half carried RNA barcode 2. After administering this AAV library to mice, performing duplex BaseScope in situ hybridization for barcode 1 and barcode 2 allowed us to quantify the number of cells that received one AAV and the number that received multiple AAVs from the library (Fig. 3). Using RNA barcodes to directly quantify the coinfection rate of the experimental AAV library, rather than estimating the potential coinfection rate by mixing reporter AAVs, can definitively identify the optimal dose that maximizes the number of cells that receive only a single AAV from the pooled library. In addition, the single-transcript sensitivity of BaseScope in situ hybridization ensures that the coinfection rate is not underestimated due to insufficient expression of reporter proteins for immunolabeling. RNA barcodes are essential for this approach: because pooled AAV libraries package many distinct genomes, it is not typically possible to design an RNA probe that labels exactly half of the AAVs in a pooled library and a second RNA probe that labels the other half. Because all three RNA barcodes in this study are smaller than 45 bases, they can be readily added to any pooled AAV library, providing a simple and validated method to quantify the coinfection rate at various doses. Although in this study we delivered an AAV2retro library to corticospinal neurons by injection into the spinal cord, this general approach of multiplexing two RNA barcodes in a pooled AAV library is compatible with any AAV capsid or route of delivery.

RNA barcodes can also improve the rigor and reliability of intersectional experiments that use multiple AAV vectors to manipulate specific neuron populations defined by their axonal connectivity (Haggerty et al., 2020; Weinholtz and Castle, 2021). Such intersectional approaches often combine an “AAV-Cre” vector encoding Cre recombinase with a “Cre-dependent AAV” carrying Cre-recognition “lox” sites. When both AAVs transduce the same cell, Cre expressed by AAV-Cre recombines the genome of the Cre-dependent AAV, resulting in gene expression only in cells that receive both vectors. Delivering AAV-Cre by axonal transport can thus specifically activate gene expression only in neurons that innervate or receive input from a defined region (Tervo et al., 2016; Zingg et al., 2017). This approach can be highly effective but may be biased by mistargeted AAV injections, by off-target recombination of the Cre-dependent genome in the absence of Cre, or by “leaky” transcription of the Cre-dependent transgene without recombination by Cre (Fischer et al., 2019). These potential confounds often cannot be monitored by standard immunolabeling techniques: for example, because Cre-dependent AAV does not typically translate any protein before recombination by Cre, it is not possible to examine the accuracy and distribution of Cre-dependent AAV injections by immunolabeling.

In this study, we added multiple RNA barcodes to a Cre-dependent AAV9-FLEX-tdTomato vector: barcode 1 is present on the AAV mRNA prior to recombination by Cre, and thus labeling barcode 1 can track the accuracy of injections and the distribution of this Cre-dependent AAV within the brain (Fig. 4*A*). Barcode 2 is present on the AAV mRNA after recombination by Cre, and thus labeling barcode 2 can identify cells that received both AAVs, resulting in recombination of the Cre-dependent AAV genome and intersectional expression of tdTomato (Fig. 4*A*). We also added barcode 3 to the AAV-Cre mRNA: labeling mRNA transcripts from both AAV-Cre and the Cre-dependent AAV can track the distribution of both injections, which may be essential for interpreting results and optimizing the experimental design. For example, after injection of AAV9-FLEX-tdTomato into mouse sensorimotor cortex and injection of AAV2retro-Cre into the cervical spinal cord, we observed intersectional expression of tdTomato in layer 5 corticospinal neurons (Fig. 4). By labeling barcode 3 on the AAV2retro-Cre mRNA, we found that AAV-Cre transduction spread to additional layer 5 cortical neurons located both rostral and caudal of the Cre-dependent AAV9-FLEX-tdTomato injection site (Fig. 4*B*). This clearly indicates that performing additional injections of the Cre-dependent AAV could engage a greater number of corticospinal neurons in this experiment.

Barcode 2 labeling could also potentially reflect leaky transcription of the Cre-dependent sequence in the absence of Cre (Fig. 4). After transduction, AAV genomes persist in the nucleus as episomal double-stranded DNA (Afione et al., 1996). Prior to recombination by Cre, the Cre-dependent tdTomato and barcode 2 sequences are present on the AAV genome in reverse (antisense) orientation (Fig. 4*A*). Thus, if transcription of the antisense strand is initiated from the 3′ end of the AAV vector (rather than transcription of the sense strand initiated by the promoter at the 5′ end of the AAV vector), this will produce an mRNA molecule that contains the tdTomato and barcode 2 sequences without recombination of the AAV genome (Fischer et al., 2019). Although leaky transcription of the antisense strand could potentially bias results, colabeling for barcode 3 on the AAV-Cre mRNA can determine whether barcode 2 labeling represents on-target transcription of the sense strand from recombined AAV genomes or off-target transcription of the antisense strand from unrecombined AAV genomes. If no barcode 3 labeling of AAV-Cre mRNA is observed in cells that are labeled by barcode 2, the latter likely indicates leaky transcription of the antisense strand in the absence of Cre (Fig. 4*B*). In this experiment, the Cre-dependent AAV9-FLEX-tdTomato vector used an “ATG-out” design: the tdTomato coding sequence on the antisense strand lacked an ATG start codon, and a single ATG start codon was instead placed on the sense strand between the promoter and lox sites (Fischer et al., 2019). This ensures that leaky transcription of the antisense strand does not result in translation of tdTomato and that tdTomato is produced only after Cre flips the tdTomato sequence into the same sense orientation as the ATG start codon. This explains why tdTomato immunolabeling was restricted to layer 5 corticospinal neurons in this experiment, despite extensive leaky transcription of the antisense strand indicated by barcode 2 labeling in other cortical layers that did not receive AAV-Cre (Fig. 4*B*).

When performing in situ hybridization for AAV mRNA, researchers should account for possible off-target labeling of AAV episomal DNA. Because the AAV DNA genome will carry the same barcode sequence as the mRNA transcript, unmasking of AAV DNA can result in off-target hybridization of an RNA probe to the DNA genome. However, because most AAV mRNAs are cytoplasmic and AAV DNA genomes are tightly confined to the nucleus, such off-target labeling can be visually identified: AAV mRNAs are labeled as discrete individual puncta throughout the cytoplasm (Figs. 1–3), while AAV DNA labeling is densely packed within the nucleus (Extended Data Fig. 4-1). In this experiment, dense nuclear labeling of AAV DNA was observed only after a relatively high concentration of AAV9 (5 × 10^12^ vg/ml) was directly injected into the brain, suggesting that DNA labeling may occur when a high multiplicity of infection results in a relatively large number of AAV DNA episomes within a single nucleus. If dense nuclear labeling is observed, protocols can be optimized to prevent unmasking of AAV DNA. We found that reducing the target retrieval duration from 15 to 10 min at 99°C largely eliminated DNA labeling while preserving on-target labeling of AAV mRNA (Extended Data Fig. 4-1). It may also be possible to reduce unmasking of AAV DNA by decreasing the temperature of target retrieval, for example, from 99 to 95°C (Baker et al., 2017). Alternatively, AAV DNA may be removed from tissue sections by DNase treatment prior to BaseScope in situ hybridization (Extended Data Fig. 4-2). If off-target labeling of AAV DNA is problematic, we recommend performing DNase treatment to eliminate DNA labeling (see Extended Data Figs. 1-1, 3-1 for step-by-step protocols).

Although RNA barcodes support the sensitive and flexible labeling of AAV mRNA, this approach has several key limitations. Small RNA barcode sequences are challenging to detect by standard in situ hybridization methods (Young et al., 2020). In this study, we used the BaseScope in situ hybridization platform, which can detect RNA sequences as small as 40 bases (Baker et al., 2017). However, the BaseScope platform is not compatible with fluorescence and can only be used for chromogenic labeling. In addition, BaseScope can only label a maximum of two RNA probes on the same tissue section, and chromogenic BaseScope labeling cannot readily be combined with immunolabeling on the same section. These limitations can be addressed by performing fluorescent in situ hybridization (Young et al., 2020). For example, the RNAscope in situ hybridization platform supports multiplexed fluorescent labeling of several probes, and RNAscope staining can be combined with fluorescent immunolabeling on the same tissue section (Wang et al., 2012; Ball et al., 2023). Although RNAscope requires a minimum target sequence of 300 bases, future studies could develop larger 300-base RNA barcodes for fluorescent RNAscope staining, although the use of these larger barcodes would be constrained by the limited packaging capacity of the AAV genome.

In conclusion, we validated three RNA barcodes for sensitive histological labeling of AAV mRNA across different species, AAV vectors, and routes of administration. These small RNA barcodes can be readily added to any AAV genome, and we provide detailed step-by-step protocols for BaseScope in situ hybridization labeling of barcoded AAV mRNA (Extended Data Figs. 1-1, 3-1). These barcodes can be multiplexed in pooled AAV libraries to determine the coinfection rate with maximum sensitivity and without the need for reporter proteins. RNA barcodes can also be multiplexed to track the distribution of multiple AAVs and the recombination of Cre-dependent AAV genomes, and to discriminate between on-target intersectional expression and off-target leaky transcription from Cre-dependent AAVs in the absence of Cre. These small RNA barcodes for multiplexed labeling of AAV transcripts will improve the consistency and reproducibility of modern neuroscience research.

## Synthesis

Reviewing Editor: Jennifer Dulin, Texas A&M University Decisions are customarily a result of the Reviewing Editor and the peer reviewers coming together and discussing their recommendations until a consensus is reached. When revisions are invited, a fact-based synthesis statement explaining their decision and outlining what is needed to prepare a revision will be listed below. The following reviewer(s) agreed to reveal their identity: NONE.

This manuscript describes a flexible and potentially useful RNA barcoding strategy for multiplexed detection of AAV-transduced cells across species, with clear advantages for pooled AAV screening experiments. The current presentation is largely qualitative and lacks sufficient quantitative validation, including barcode design rationale, specificity, and efficiency metrics. In addition, the study does not adequately address a key complication of AAV biology (the distinction between viral DNA and RNA) relying primarily on localization and protocol optimization rather than definitive RNase/DNase or strand-specific controls. Addressing these methodological limitations and more explicitly discussing the scope and constraints of the approach will strengthen the manuscript.

-----

Reviewers' comments:

Reviewer #1:

Major concern:

Over the results are presented in a very qualitative manner and it lacks quantitative validation. There are very few number attesting the efficacy of the method. Furthermore the authors provide three different barcodes. They don't indicate how they were designed and do not provide any specificity data between different barcodes.

Minor concerns:

Line 150, complete [author university]. Same in line 194

Line 93, what is smFLAG

Line 107, could you please give more information about the 110 bp synthetic intron. The provided reference is only about the improved Cre.

Reviewer #2:

The manuscript presents a useful RNA barcoding strategy for multiplexed detection of AAV transcripts, which is particularly advantageous for small pooled AAV screening experiments. However, AAV biology presents a unique complication in this context, as the viral genome can exist as single-stranded and/or double-stranded DNA depending on the stage after infection. Therefore, it is critical to distinguish between DNA copies and RNA transcripts when interpreting RNAscope signals. In practice, one approach would be to include DNase pretreatment controls to reduce potential DNA-derived hybridization. However, because AAV genomes persist as episomes, they may exhibit resistance to DNase treatment, which adds complexity to data interpretation.

Anyway, incorporating RNase and DNase controls would still be valuable to enhance specificity, clarify signal origin. in this manuscript, however, the distinction between AAV RNA and DNA relies primarily on subcellular localization patterns and optimization of heat-induced target retrieval. The study would be strengthened by incorporating additional controls to more definitively differentiate RNA from DNA signals, such as RNase (or DNase) pretreatment experiments. Including these controls would increase confidence that the reported signals reflect true transcription rather than hybridization to viral DNA, eg. under high viral dose conditions. The reviewer suggested to include Rnase (and Dnase) pretreatment controls. It is also useful to include strand specific probing.it is important to further discuss the limitations of the methods.

## Author Response

RE: "Small RNA barcodes for sensitive labeling of AAV transcripts" (eN-MNT-0034-26) Dear Dr. Dulin, We thank the reviewers for their helpful comments, which have improved the manuscript. We address each comment below.

Reviewer #1 1) "The results are presented in a very qualitative manner and it lacks quantitative validation. There are very few number attesting the efficacy of the method." We performed a quantitative experiment to compare the sensitivity of GFP immunolabeling and barcode 1 RNA labeling for quantifying distal transduction in thalamus after injection of barcoded AAV9-sfGFP into mouse sensorimotor cortex. These new results are shown in Fig.

1B,C and are described in lines 243-254 and 291-302. BaseScope labeling of barcoded AAV mRNA was significantly more sensitive than GFP immunolabeling for detecting transduced cells in thalamus.

We also quantified the number of transduced cells in rhesus macaque spinal cord detected by BDNF immunolabeling or by barcode 1 RNA labeling after infusion of scAAV9-BDNF into the cerebrospinal fluid. In this experiment, BDNF immunolabeling cannot conclusively determine the rate of transduction, because immunolabeling may also detect endogenous BDNF protein, and cells transduced by AAV9 may secrete BDNF onto neighboring cells. These new results are shown in Fig. 2B and described in lines 255-261 and 308-316. Both immunolabeling and BaseScope in situ hybridization labeled similar numbers of transduced cells. Barcode 1 labeling of AAV mRNA can thus ensure accurate quantification of AAV transduction when the AAV transgene product is secreted or is identical to an endogenous protein.

2) "The authors provide three different barcodes. They don't indicate how they were designed and do not provide any specificity data between different barcodes." We added a detailed description of the barcode design to lines 276-282. RNA barcodes were sized between 40 and 44 bases to be compatible with a single 1ZZ BaseScope probe pair. The barcodes did not contain biologically active sequences such as start codons, CpG motifs, or consensus sequences for intron splicing or transcription initiation. RNA barcode probes were not predicted to cross-react with any known mammalian genome or RNA sequences by alignment search against taxID 40674 (mammals), with any known viral genome or RNA sequences (taxID 10239, viruses), or with any common synthetic sequences (taxID 32630, synthetic constructs).

We performed additional labeling to examine the specificity of each probe in matched brain sections from mice that received AAV injection into motor cortex. These new results are shown in Fig. 5 and described in lines 422-426. Matched sections were stained with each BaseScope barcode probe under identical conditions. Each probe specifically reacted with only the target RNA barcode, and did not cross-react with other barcodes.

3) Minor concern: "Minor concerns: Line 150, complete [author university]. Same in line 194" This information was removed in accordance with the journal's double-blind review policy. Identifying information will be included in the clean copy of the revised manuscript that is submitted for publication.

4) Minor concern: "Line 93, what is smFLAG" We added this information to the Methods, lines 98-99. The smFLAG reporter is an engineered variant of mRuby2 with ten FLAG epitopes for immunolabeling (Viswanathan et al., 2015).

5) Minor concern: "Line 107, could you please give more information about the 110 bp synthetic intron. The provided reference is only about the improved Cre." We added this information and the complete synthetic intron sequence to the Methods, lines 106-110. The synthetic intron is a minimal intron that combines strong mammalian consensus splice donor, splice acceptor, and branch point sequences.

Reviewer #2 1) "The study would be strengthened by incorporating additional controls to more definitively differentiate RNA from DNA signals, such as RNase (or DNase) pretreatment experiments... It is also useful to include strand specific probing." We performed a new experiment to selectively remove DNA or RNA from tissue sections by DNase or RNase treatment prior to BaseScope in situ hybridization. For strand specific probing, we performed this experiment in mice that received the same Cre-dependent AAV9- FLEX-tdTomato vector used in Fig. 4, but that did not receive an AAV-Cre vector. In the absence of Cre, few AAV genomes are expected to recombine: nearly all AAV mRNA should be transcribed from the top strand (barcode 1, green), and minimal AAV mRNA should be transcribed from the bottom strand (barcode 2, red). These new results are shown in Extended Data Fig. 4-2 and described in lines 405-421. Strong cytoplasmic labeling of AAV mRNA transcribed from the top strand (barcode 1, green) was observed surrounding the cortical injection site, along with dense nuclear labeling of both barcodes consistent with off- target labeling of AAV DNA episomes. After RNase treatment, cytoplasmic labeling of AAV mRNA was greatly reduced (barcode 1, green), while the dense nuclear labeling was not visibly decreased. In contrast, DNase treatment almost completely eliminated the dense nuclear labeling. Minimal labeling of barcode 2 (red) was observed after DNase treatment. This provides further evidence that nuclear labeling was caused by off-target probe binding to AAV DNA episomes, and demonstrates that off-target DNA labeling may be prevented by DNase treatment prior to BaseScope in situ hybridization.

We also added these methods for DNase and RNase treatment to the step-by-step protocols provided as Extended Data Fig. 1-1 and Extended Data Fig. 3-1.

2) "It is important to further discuss the limitations of the methods." We added a discussion of limitations to lines 567-581. Small RNA barcode sequences are challenging to detect by standard in situ hybridization methods. In this study, we used the BaseScope in situ hybridization platform, which can detect RNA sequences as small as 40 bases. However, the BaseScope platform is not compatible with fluorescence and can only be used for chromogenic labeling. In addition, BaseScope can only label a maximum of two RNA probes on the same tissue section, and chromogenic BaseScope labeling cannot readily be combined with immunolabeling on the same section. These limitations can be addressed by performing fluorescent in situ hybridization. For example, the RNAscope in situ hybridization platform supports multiplexed fluorescent labeling of several probes, and RNAscope staining can be combined with fluorescent immunolabeling on the same tissue section. Although RNAscope requires a minimum target sequence of 300 bases, future studies could develop larger 300-base RNA barcodes for fluorescent RNAscope staining, although the use of these larger barcodes would be constrained by the limited packaging capacity of the AAV genome.

We are grateful for the reviewers' comments, and we hope that our responses adequately address all points.

Sincerely, Michael J. Castle, Ph.D.

Assistant Professor of Neurosciences University of California, San Diego
