# Supplementary material for: Small RNA Barcodes for Sensitive Labeling of AAV Transcripts
Source: eNeuro. 2026 Aug 4;13(8):ENEURO.0034-26.2026. doi: 10.1523/ENEURO.0034-26.2026 (PMC13446960; doi:10.1523/ENEURO.0034-26.2026)
Supplement: Supplementary file 1 [file eneuro-13-ENEURO.0034-26.2026-s002.docx]

**Materials and Reagents**

- BaseScope Red reagent kit v2 (ACD #323900), containing:
  - BaseScope detection reagents v2 RED (ACD #323910)
  - RNAscope H_2_O_2_ and protease reagents (ACD #322381)
  - RNAscope target retrieval reagents (ACD #322000)
  - RNAscope wash buffer reagents (ACD #310091)
- 1ZZ **Channel 1** BaseScope probe, **Barcode 1** (ACD #902371, BA-Barcode-sequence-8-1zz-st)
  - Target sequence: CCCAGTCAGCAGCCCTGTTCACTGCCCTAAGCCCTCTAGC
- 1ZZ **Channel 1** BaseScope probe, **Barcode 2** (ACD #902341, BA-Barcode-sequence-7-1zz-st)

Target sequence: AATTACAAGCCTACCTGTGGCTAATAGGCCAAGCAGTTGTTGGT

- 1ZZ **Channel 1** BaseScope probe, **Barcode 3** (ACD #1174221-C1, BA-Syn-BC10-1zz-st-C1)
  - Target sequence: TGACTTTGGCAATCCCTTGTGTCTCTCTGTATAGGCTGGCTCAA
- 1ZZ **Channel 1** BaseScope negative control probe, DapB (ACD #701021, BA-DapB-1ZZ)
- 1ZZ **Channel 1** BaseScope positive control probe, **mouse** Ppib (ACD #712351, BA-Mm-Ppib-1zz)
  - Positive control probes for other species are also available from ACD
- VectaMount Coverslipping Medium (Vector Laboratories #H-5700-60)
  - Alternative: EcoMount Coverslipping Medium (Biocare Medical #EM897L)
- 1X (0.01 M) phosphate-buffered saline (PBS) or 1X (0.1 M) tris-buffered saline (TBS), pH 7.4
- Tween-20 (polysorbate 20)
- Freshly prepared 4% paraformaldehyde (PFA) in 0.1 M phosphate buffer, pH 7.4
- 100% ethyl alcohol (ethanol)
- Sodium chloride (NaCl)
- Sodium citrate dihydrate
- Hematoxylin
- Clarifier 2 solution
- Bluing solution
- Xylene

**Equipment**

- HybEZ hybridization oven (ACD #321710/321720)
- HybEZ humidity control tray with lid (ACD #310012)
- EZ-Batch Slide Holder, 20 slide capacity (ACD #321716)
- Microscope slide staining rack with compatible staining dishes
- ImmEdge hydrophobic barrier (PAP) pen (Vector Laboratories #H-4000)
- Food steamer, such as Hamilton Beach digital food steamer, 5.5 quart, model #37530
- Convection oven, such as Cole-Parmer model #OVF-800-61-120
- Laboratory fume hood

**DAY 1: Sample Preparation**

For optimal labeling of rodent tissues, perfuse animals either with freshly prepared ice-cold 4% PFA only, or with cold, **nuclease-free** PBS followed by cold 4% PFA. If perfusion with 4% PFA is not possible, drop fix tissues in cold 4% PFA as quickly as possible after PBS perfusion (within 5 minutes). After perfusion, drop fix overnight at 4°C in 4% PFA, then transfer to 30% sucrose at 4°C. Tissues from larger species may need to be drop fixed for multiple overnights for optimal fixation.

This protocol is optimized for 30 to 40 µm-thick brain or spinal cord tissue sections.

1. For free-floating tissue sections, wash sections for ~1 min in 0.5X PBS or TBS containing 0.1% Tween-20 to remove cryoprotectant. Repeat 3 additional times in fresh buffer.
   a. For cryostat sections that are already adhered to slides, wash the slides for ~5 min in 0.5X PBS or TBS to remove embedding medium. Proceed to step 3.
2. Transfer sections to 0.5X PBS or TBS containing 0.1% Tween-20. Use a fine brush to mount sections on positively charged (“plus”) glass microscope slides. Group sections as closely together as possible. Leave sufficient space around tissue to draw a barrier.
   a. Under a dissecting microscope, use a dry brush to press the tissue down against the slide, removing all liquid and adhering the tissue to the slide. Ensure that every part of the tissue is pressed flat against the slide. Use vacuum aspiration to remove all liquid from underneath the section by carefully aspirating around the edge of the tissue on all sides. *This is essential to prevent lifting or detachment of tissue sections during pretreatment and target retrieval.*
   b. Use fresh (not expired) positively charged (“plus”) slides.
3. Dry slides for **1 hour** at room temperature (RT).
4. Bake slides overnight (at least 18 hours) at 50°C in a convection oven.

**DAY 2: Target Retrieval**

1. Place the slides in a microscope slide staining rack. Postfix the slides in freshly prepared 4% PFA for **2 hours** at RT.
2. Gently agitate the slides in 0.5X PBS or TBS containing 0.1% Tween-20 for several seconds. Repeat 2 additional times in fresh buffer.
3. Dehydrate the slides by sequentially incubating in 50%, 70%, 80%, and 95-100% ethanol at RT for 2 minutes each.
4. Dry the slides for 1 hour at ~50°C in a convection oven.
5. Let slides cool to RT. Add 5 drops of H_2_O_2_ (Pretreatment 1) to the slide. Incubate for **10 minutes** at RT. Use a timer to stagger treatment so that each slide can proceed to step 6 after exactly 10 minutes.
   a. Typical 25x75 mm slides require 4 to 5 drops of each reagent per slide, depending on the size of the barrier. Typical 50x75 mm slides require 8 to 12 drops per slide. For large barriers, it may be necessary to use a pipette tip to evenly spread the solution over the tissue. Make sure tissue remains completely immersed in liquid during every incubation.
6. Remove H_2_O_2_ by flicking the slides on absorbent paper. Wash slides in distilled water by gently agitating. Repeat wash one additional time in fresh distilled water.
7. Dry the slides for 15 minutes at 50°C in a convection oven.
8. Fill a food steamer with cold tap water to the maximum fill line**. Do not add more water during target retrieval**. If using a Hamilton Beach Digital Food Steamer (5.5 quart), stack both steaming bowls without a divider.
9. Make fresh 1X target retrieval solution (pretreatment 2) by diluting 20 mL of 10X target retrieval solution in 180 mL of distilled water in a plastic slide staining dish. Add ~200mL of distilled water to a second slide staining dish. Place both staining dishes into the steaming bowl. Place lid on the steamer and directly insert a thermometer through the lid into the target retrieval solution. Turn on the steamer.
10. When the temperature on the thermometer reaches **99°C**, place the slide rack into the distilled water and incubate for **10 seconds**. After 10 seconds, transfer the slide rack to the target retrieval solution, close the lid, and incubate for **15 minutes**.
    a. Monitor the thermometer in the target retrieval solution throughout the incubation. **Do not allow the temperature to exceed 99°C.** If the temperature exceeds 99°C, release steam by opening the lid.
    b. Prolonged target retrieval may degrade the tissue or result in labeling of AAV episomal DNA. Reduce incubation to **10 minutes** if tissue is degraded or if labeling of AAV episomal DNA is observed. Alternatively, pretreat slides with DNase to remove episomal DNA (see above).
    c. *Alternative protocol for manual target retrieval:* place a pot on a hot plate. Add water to the pot, place both staining dishes in the water, and place a thermometer in the target retrieval solution. Heat until the temperature reaches 100°C (water in the pot will start to boil). Dip the slide rack into the distilled water for 10 seconds, then transfer the slide rack to the target retrieval solution, cover with foil, and incubate for 10–15 minutes. Maintain temperature between 98–102°C and ensure that the target retrieval solution does not boil during the incubation.
11. Incubate the slides in 200 mL of distilled water for 15 seconds at RT.
12. Incubate the slides in 100% ethanol for 3 minutes at RT.
13. Dry the slides for 10 minutes at 50°C in a convection oven.

**DAY 2: Hybridization**

14. Place a damp paper towel in a humidity control tray with EZ-Batch slide holder. Cover the tray and insert it into the hybridization oven. Warm the oven to 40°C for at least 30 minutes.
15. Warm all probes for 10 minutes at 40°C, then cool to RT before use.
16. Use Vector Laboratories H-4000 ImmEdge Hydrophobic Barrier Pen to draw a rectangular barrier on each slide. Wait several seconds, then repeat 1 to 3 additional times. Allow the barrier to dry completely (at least 1 minute) before proceeding.

*Optional Control: DNase Treatment*

*To remove DNA from control slides, perform the below before proceeding to step 17:*

- Leave untreated slides dry at RT while these steps are performed.
- Wash slides 3 times, 2 minutes each in distilled water.
- Combine 10 µL of DNase-1 (Sigma #D5319) and 240 µL of DNase buffer (**1:25** dilution). Mix by inverting several times (**do not vortex**), then spin down.
  - *DNase buffer recipe*: Dissolve 10 mM TrisCl, 2.5 mM MgCl_2_, and 0.5 mM CaCl_2_ in distilled water. Adjust the pH to 7.5.
- Flick off excess liquid from the slides. Add diluted DNase-1 solution to cover each section.
- Incubate in the hybridization oven for **1 hour** at 40°C.
- Carefully remove excess DNase solution from the slides.
  - Residual DNase solution may de-stabilize probe binding. Be careful to completely remove DNase solution.
- Wash slides 3 times, 2 minutes each in distilled water.

17. Add slides to the slide holder in the humidity control tray. Add 5 drops of Protease III (for mouse/rat tissue) or Protease IV (for macaque/human tissue). Incubate in the hybridization oven for **35 minutes** at 40°C.
    a. The optimal protease (III or IV) and incubation time (15 to 45 min) depends on the tissue and fixation. For 40 µm-thick sections fixed in 4% PFA, 35 minutes is optimal. For thinner sections use a shorter incubation time. If tissue is underdigested (weak signal or nonspecific labeling), extend incubation time. If tissue is overdigested (perforated tissue and degraded cell morphology in hematoxylin stain), reduce incubation time.
    b. **For the remainder of the protocol work quickly and do not allow the slides to completely dry.**
18. Remove slides from hybridization oven and flick off excess liquid. Wash slides in distilled water by gently agitating. Repeat wash one additional time in fresh distilled water.

*Optional Control: RNase Treatment*

*To remove RNA from control slides, perform the below before proceeding to step 19:*

- Leave untreated slides in distilled water while these steps are performed.
- Perform RNase treatment in a dedicated location where no RNA experiments are performed. Be careful to avoid cross-contamination of reagents or equipment with RNase solution.
- Combine 10 µL of 100 mg/mL RNase A (Qiagen #19101) and 190 µL of 1X PBS (**1:20** dilution). Mix by briefly vortexing, then spin down.
- Flick off excess liquid from the slides. Add diluted RNase solution to cover each section.
- Incubate in the hybridization oven for **30 minutes** at 40°C.
- Carefully remove excess RNase solution from the slides. Be careful to completely remove all RNase solution and to avoid cross-contamination of untreated slides.
- Wash slides 3 times, 2 minutes each in distilled water.
- Clean the work area and any equipment that may have contacted the RNase solution with RNaseZap decontamination solution (Sigma #R2020).

19. Flick off excess water from the slides. Add 120 µL of channel 1 probe to each section.
20. Incubate slides in the hybridization oven for 2 hours at 40°C.
21. Remove slides from the hybridization oven. Wash slides by gently agitating in 1X wash buffer for 5 minutes at RT. Wash two additional times with fresh 1X wash buffer.
    a. To prepare wash buffer, warm 50X wash buffer to 40°C for 10 to 20 minutes. Combine 2.94 L of distilled water with a full bottle (60 mL) of 50X wash buffer in a large Erlenmeyer flask. Mix well. 1X wash buffer can be stored at RT for up to 1 month. Mix well before each use.
22. Transfer slides to 5X SSC and incubate overnight at RT.
    a. To prepare 500 mL of 5X SSC, combine 125 mL of 20X SSC with 375 mL of distilled water.
    b. Recipe for 20X SSC:
       i. Dissolve 175.3 g of NaCl and 88.2 g of sodium citrate dihydrate in 800 mL of distilled water.
       ii. Adjust the pH to 7.0.
       iii. Bring the volume to 1 L with distilled water.
       iv. Sterilize by autoclaving.
       v. Store autoclaved 20X SSC at room temperature for up to 6 months.

**DAY 3: Amplification**

1. Place a damp paper towel in a humidity control tray with EZ-Batch slide holder. Cover the tray and insert it into the hybridization oven. Warm the oven to 40°C for at least 30 minutes.
2. Place AMP 1-8 at RT for at least 30 minutes before use. Return each vial to 4°C after use.
3. Wash the slides in 1X wash buffer for 2 minutes at RT. Gently agitate the slides. Wash one additional time in fresh 1X wash buffer.
4. Flick off excess liquid from the slides and add ~4 drops of AMP **1**. Incubate for **30** **minutes** at 40°C in the hybridization oven.
5. Remove slides from the oven and flick off excess liquid. Wash slides in 1X wash buffer for 2 minutes at RT. Gently agitate the slides. Wash one additional time in fresh 1X wash buffer.
6. Flick off excess liquid from the slides and add ~4 drops of AMP **2**. Incubate for **30** **minutes** at 40°C in the hybridization oven.
7. Remove slides from the oven and flick off excess liquid. Wash slides in 1X wash buffer for 2 minutes at RT. Gently agitate the slides. Wash one additional time in fresh 1X wash buffer.
8. Flick off excess liquid from the slides and add ~4 drops of AMP **3**. Incubate for **15** **minutes** at 40°C in the hybridization oven.
9. Remove slides from the oven and flick off excess liquid. Wash slides in 1X wash buffer for 2 minutes at RT. Gently agitate the slides. Wash one additional time in fresh 1X wash buffer.
10. Flick off excess liquid from the slides and add ~4 drops of AMP **4**. Incubate for **30** **minutes** at 40°C in the hybridization oven.
11. Remove slides from the oven and flick off excess liquid. Wash slides in 1X wash buffer for 2 minutes at RT. Gently agitate the slides. Wash one additional time in fresh 1X wash buffer.
12. Flick off excess liquid from the slides and add ~4 drops of AMP **5**. Incubate for **30** **minutes** at 40°C in the hybridization oven.
13. Remove slides from the oven and flick off excess liquid. Wash slides in 1X wash buffer for 2 minutes at RT. Gently agitate the slides. Wash one additional time in fresh 1X wash buffer.
14. Flick off excess liquid from the slides and add ~4 drops of AMP **6**. Incubate for **15** **minutes** at 40°C in the hybridization oven.
15. Remove slides from the oven and flick off excess liquid. Wash slides in 1X wash buffer for 2 minutes at RT. Gently agitate the slides. Wash one additional time in fresh 1X wash buffer.
16. Remove the humidity control tray and turn off the hybridization oven.
17. Flick off excess liquid from the slides and add ~4 drops of AMP **7**. Incubate for **30** **minutes** at **RT** in the humidity control tray.
    a. To increase the intensity of the red signal, incubate for **60** **minutes** at RT.
18. Remove slides from the tray and flick off excess liquid. Wash slides in 1X wash buffer for 2 minutes at RT. Gently agitate the slides. Wash one additional time in fresh 1X wash buffer.
19. Flick off excess liquid from the slides and add ~4 drops of AMP **8**. Incubate for **15** **minutes** at **RT** in the humidity control tray.
    a. If the Amp 7 incubation (step 17) was for 60 minutes, incubate for **30** **minutes** at RT.
20. Remove slides from the tray and flick off excess liquid. Wash slides in 1X wash buffer for 2 minutes at RT. Gently agitate the slides. Wash one additional time in fresh 1X wash buffer.

**DAY 3: Signal Detection**

21. Spin down RED-B tube. Add 2 µL RED-B to 120 µL RED-A in an Eppendorf tube (**1:60** dilution). The solution should become pink (do not use the solution if it remains clear). Do not expose the mixed RED solution to sunlight or UV light. Vortex briefly and use within 5 minutes.
22. Flick off excess liquid from slides, then add 120 µL RED solution to each slide. Incubate for **10** **minutes** at RT in the humidity control tray. **DO NOT EXCEED 10 MINUTES.**
23. Remove slides from the tray and flick off excess liquid. Wash slides in 1X wash buffer for 2 minutes at RT. Gently agitate the slides. Wash one additional time in fresh 1X wash buffer.
24. Do not expose the slides to ethanol after signal detection is complete.

**DAY 3: Counterstain (Optional)**

25. Immerse the slide rack in distilled water.
26. Place a slide rack in a staining dish under running tap water. Pick up one slide at a time and flick off as much water as possible. Dip the slide in and out of 50% hematoxylin two times in a continuous movement (~2 seconds). Immediately place the slide in the slide rack under running tap water. Tilt the staining dish to run fresh tap water across the slide until all hematoxylin is removed. Transfer the slide to a slide rack immersed in fresh tap water. Repeat for all slides. Do not run tap water directly onto the slides.
    a. To prepare 50% hematoxylin, combine 100 mL of distilled water with 100 mL of hematoxylin. Mix thoroughly.
    b. To intensify the counterstain, extend the hematoxylin incubation.
27. Immerse the slide rack in Clarifier 2 solution. Gently agitate the slides in Clarifier 2 solution and incubate for 30 seconds at RT.
28. Immerse the slide rack in tap water. Gently agitate the slides under running tap water for at least 30 seconds. Do not run tap water directly onto the slides.
29. Immerse the slide rack in Bluing solution. Gently agitate the slides in Bluing solution and incubate for 1 minute at RT.
30. Immerse the slide rack in tap water. Gently agitate the slides under running tap water for at least 1 minute. Do not run tap water directly onto the slides.

**DAY 3: Coverslipping**

31. Dry the slides for 60 minutes at 50°C in a convection oven.
32. Cool the slides for 5 minutes at RT.
33. Perform coverslipping in a fume hood. Quickly dip each slide in Xylene for ~1-2 seconds, flick off excess, and immediately proceed to the next step.
34. Place 1-2 drops of VectaMount or EcoMount coverslipping medium on the slide. Carefully place a coverslip over the tissue section. Dry slides overnight at RT.
    a. Image slides within 2 weeks.
